# The Role of Exercise as a Non-pharmacological Therapeutic Approach for Amyotrophic Lateral Sclerosis: Beneficial or Detrimental?

**DOI:** 10.3389/fneur.2019.00783

**Published:** 2019-07-17

**Authors:** Stavroula Tsitkanou, Paul Della Gatta, Victoria Foletta, Aaron Russell

**Affiliations:** School of Exercise and Nutrition Sciences, Institute for Physical Activity and Nutrition, Deakin University, Geelong, VIC, Australia

**Keywords:** amyotrophic lateral sclerosis/ALS, motor neuron disease, exercise, endurance training, resistance training, SOD1^*G*93*A*^ mice, ALS patients

## Abstract

Amyotrophic lateral sclerosis (ALS), a fatal neurodegenerative disease, involves the rapid deterioration of motor neurons resulting in severe muscle atrophy and respiratory insufficiency. It is considered a “multisystemic” disease with many potential mechanisms responsible for its pathology. Currently, there is no cure for ALS. Exercise training is suggested as a potential approach to reduce ALS pathology, but its beneficial role remains controversial. This review provides an overview of the effects of exercise training in ALS-affected mice and patients. It will compare the intensity, duration, and type of exercise on the health of SOD1^G93A^ mice, a mouse model of familial ALS, and review clinical studies involving ALS patients undergoing both endurance and resistance training. In summary, mild-to-moderate swimming-based endurance training appears the most advantageous mode of exercise in SOD1^G93A^ mice, improving animal survival, and delaying the onset and progression of disease. Furthermore, clinical studies show that both endurance and resistance training have an advantageous impact on the quality of life of ALS patients without extending life expectancy. However, small sample sizes, non-representative control populations, heterogeneous disease stage of patients, and the presence of confounders often exist in the exercise studies conducted with ALS patients. This raises concerns about the interpretation of these findings and, therefore, these results should be considered with caution. While promising, more pre-clinical and clinical studies with improved experimental design and fewer limitations are still necessary to confirm the impact of exercise training on the health of ALS patients.

## Introduction

Amyotrophic lateral sclerosis (ALS), a subtype of fatal motor neuron disease (MND), is a progressive and degenerative neuromuscular disorder. It is characterized by the death of the upper and lower motor neurons at the spinal or bulbar level, leading to paralysis and eventual death usually from severe muscle atrophy and respiratory insufficiency. Median survival is between 2 and 4 years from the onset of symptoms (1). The disease usually appears between 40 and 70 years of age, and affects about five in 100,000 people worldwide (2). There are two types of ALS: a familial ALS (FALS), heritable form (~5–10% of ALS), and the sporadic ALS (SALS) form, which occurs randomly (~90–95% of ALS). Mutations in the human copper/zinc (CuZn) superoxide dismutase (SOD1) gene account for ~20% of FALS cases, and the mutant SOD1^G93A^ rodent model has been used extensively to help understand ALS pathology and to test potential therapeutic treatments for this disease. Several factors are suggested to contribute to motor neuron death in ALS. Excitotoxicity (3, 4), deficits in axonal transport (5, 6) and neurofilament aggregation (7, 8) are neuron-related mechanisms potentially involved in pathophysiology of ALS. Non-neuronal defects, including oxidative stress (9, 10), protein misfolding/aggregation (11), neuroinflammation caused by non-neuronal cells (e.g., glial cells, astrocytes) (12, 13), and skeletal muscle dysfunction (14–16) resulting from disequilibrium in mitochondria function (17, 18), satellite cell (SC) activity (19, 20) and microRNA expression (16, 21), are also considered potential contributors to ALS pathophysiology. Therefore, ALS is considered a “multisystemic” disease, where changes in different cell types may act mutually and synergistically to contribute to disease onset and severity (22). Given the complexity of the disease, it is difficult to find a specific treatment for ALS patients. Currently, there is no effective pharmacological therapy for ALS. Interestingly, non-pharmacological approaches, such as non-invasive ventilation, have been suggested to prolong the survival in ALS patients (23, 24).

Physical activity is considered as a non-pharmacological therapeutic intervention for many diseases. In the general population, exercise training induces beneficial adaptations in both cardiovascular and neuromuscular systems, including improvements in respiratory and heart function (25), capillary density increase (26), muscle hypertrophy and muscle strength/power increase (27). In addition, it promotes psychological well-being by decreasing depression and anxiety levels, and positively influencing mood state, self-esteem and self-image (28, 29). However, exercise as an approach to reduce ALS pathology is controversial. Previous epidemiological studies suggest that a lifestyle involving vigorous exercise (30–32) and reduced body fat (32) are associated with an increased incidence of ALS. Also, there are studies which suggest an increased risk for ALS among professional soccer and American football players (33, 34). This association is physiologically plausible as strenuous and prolonged exercise may induce oxidative stress, glutamate excitotoxicity and increased calcium loads, which promote selective degeneration of vulnerable motor neurons (35, 36). On the other hand, several neurotrophic factors are up-regulated by exercise in neural and muscle tissue including brain-derived neurotrophic factor (37), which promotes survival and growth of spinal cord cells (38, 39) and glial cell-derived neurotrophic factor (40), which causes synaptic remodeling and hyperinnervation of neuromuscular junctions (41). Based on these findings, the hypothesis that physical activity is a risk factor for developing ALS seems unlikely. Instead, it has been suggested that genetic profiles modified by exogenous factors, which promote physical fitness and raise ALS susceptibility, might be a more credible explanation for the association between physical activity and ALS (17). The discordant results among the epidemiological studies of ALS can be explained by the methodological limitations inherent in studying a relatively low incidence disease. Specifically, most of these studies did not entirely account for potential confounders, such as trauma, possible use of drugs, drugs of abuse, dietary supplements, exposure to environmental toxins such as pesticides or characteristics of physical activity (mode, frequency, duration, intensity) (42, 43).

Multiple studies in ALS mice and patients have been performed to assess the effect of exercise training on disease progression. In preclinical studies, data are conflicting as exercise has been suggested to be beneficial (44), null (45) or even harmful (46) in transgenic SOD1^G93A^ mice. Similarly, in humans, there are studies supporting the benefit of exercise training on the quality of life for ALS patients (47, 48), while other studies concluded that exercise had negative outcomes on neuromuscular function of ALS patients (49). Irrespective of these findings, physical activity is still considered a potential therapeutic approach for ALS improving body function, slowing disease progression and lessening caregiver burden (50). Apart from the biological and mental improvements observed after exercise, physical activity also activates specific mechanisms disrupted during the progression of ALS. For example, exercise evokes signaling pathways modifying skeletal muscle metabolism and triggers structural myofiber remodeling through SC activation (51). Regardless of mode (endurance, resistance or concurrent training), regular exercise lowers basal oxidative stress and increases anti-oxidative capacity (52–54). Moreover, endurance exercise potently increases skeletal muscle mitochondrial content (55, 56), neurogenesis (57, 58) and has a neuroprotective effect on the brain protecting against ischemic neuronal damage in the hippocampal formation (59).

Although evidence supports the advantageous impact of exercise in ALS, it is still not clear whether physical activity should be recommended as a treatment option for ALS patients. Even if exercise training *per se* is not a sufficient intervention to decelerate disease progression or improve patients' survival, it could be an adjunct therapy to other pharmacological treatments of ALS. Considering the lack of treatments at this time in ALS, non-pharma therapeutics, such as physical activity, is likely to be more important than previously appreciated. This review highlights our knowledge of the role of exercise as a non-pharmacological therapeutic approach for ALS. More specifically, we have critically reviewed both pre-clinical animal and clinical studies to understand whether physical activity could improve the quality of life and life expectancy in ALS, as well as which type of exercise elicits greater compliance and efficacy in ALS patients. While we acknowledge the importance of alternative training methods, such as respiratory muscle training and range-of-motion exercises/stretching, used mainly by respiratory therapists, physiotherapists and occupational therapists to improve physical performance (e.g., respiratory function and flexibility), this review is focused on specific physical activity modes (endurance, resistance, and concurrent training) prescribed by exercise physiologists.

## Effects of Exercise in SOD1^**G93A**^ ALS Mice

Pre-clinical studies in rodents have assessed the effect of exercise on ALS pathophysiology. Most of them suggest mild-to-moderate endurance exercise as a positive treatment (44, 60–63); whereas, vigorous endurance training seems to have null (45) or harmful (46, 61) effects in SOD1^G93A^ ALS mice (Tables 1, 2). Compared to running-based training, swimming seems to be more beneficial in SOD1^G93A^ mice (64, 65). Therefore, the exercise mode, intensity and duration are likely critical factors in eliciting potential positive effects from endurance training in ALS mice. Interestingly, none of the exercise studies in ALS mice is resistance-based, although resistance training is suggested as a major type of exercise in many disorders, including neuromuscular diseases (66).

**Table 1:** Summary of the effects of running-based endurance training in SOD1^G93A^ ALS mice.

**Study**	**Sex**	**Age (days)**	**Duration**	**Exercise protocol**	**Various outcomes**
Kirkinezos et al. (60)	M (*n* = 15)	49	70 days	Treadmill running at 13 m/min for 30 min, 5 days/week	↑ Survival
	F (*n* = 15)	49	70 days	Treadmill running at 13 m/min for 30 min, 5 days/week	↔ Survival
Veldink et al. (44)	M (*n* = 13) Low-copy SOD1^G93A^	56	Scores >15 points (3 points for each electrical shock, 1 point after resting for 5 s)	Treadmill running at 16 m/min for 45 min, 5 days/week	↔ Onset of disease ↔ Survival
	F (*n* = 11) Low-copy SOD1^G93A^	56	Scores >15 points (3 points for each electrical shock, 1 point after resting for 5 s)	Treadmill running at 16 m/min for 45 min, 5 days/week	↑ Onset of disease ↔ Survival
	F (*n* = 9) High-copy SOD1^G93A^	56	Scores >15 points (3 points for each electrical shock, 1 point after resting for 5 s)	Treadmill running at 16 m/min for 45 min, 5 days/week	↔ Onset of disease ↑ Survival
Kassa et al. (63)	M (*n* = 5)	50	Until symptom onset	Treadmill running at 20 m/min for 45 min, 5 days/week	↔ Onset of disease ↔ Motor neuron counts
Carreras et al. (61)	M (*n*_total_ = 30, *n* = 10 for each time point)	30	40, 65, and 90 days	Moderate intensity: treadmill running at 10 m/min, for 30 min, 3 days/week	↑ Motor performance ↑ Motor neuron density
	M (*n*_total_ = 30, *n* = 10 for each time point)	30	40, 65, and 90 days	High intensity: treadmill running at 20 m/min, for 60 min, 5 days/week	↓ Motor performance ↔ Motor neuron density
Mahoney et al. (46)	M (*n* = 7)	40	A minimum of 9 m/min for 45 min	Treadmill running at 9–22 m/min for 20, 25, and 30 min for 3 days/week, the first 3 weeks, and 45 min for 5 days/week thereafter	↔ Onset of disease ↓ Survival ↓ Motor performance
	F (*n* = 7)	40	A minimum of 9 m/min for 45 min	Treadmill running at 9–22 m/min for 20, 25, and 30 min for 3 days/week, the first 3 weeks, and 45 min for 5 days/week thereafter	↔ Onset of disease ↔ Survival ↔ Motor performance
Kaspar et al. (62)	M (*n* = 9) + F (*n* = 9)	40	To right themselves within 30 s	2 h daily exposure to running wheels (3,162 revolutions/day)	↔ Motor performance ↑ Survival
	M (*n* = 9) + F (*n* = 9)	40	To right themselves within 30 s	6 h daily exposure to running wheels (5,551 revolutions/day)	↑ Motor performance ↑ Survival
	M (*n* = 9) + F (*n* = 9)	40	To right themselves within 30 s	12 h daily exposure to running wheels (10,482 revolutions/day)	↑ Motor performance ↑ Survival
Liebetanz et al. (45)	M (*n* = 7) + F (*n* = 5)	35	Speed exceeding preset wheel speed	10 h daily exposure to running wheels: 40 × 10 min of running, 5 min rest (1,400–5,000 m/day)	↔ Onset of disease ↔ Motor performance ↔ Survival
Deforges et al. (64)	M (*n* = 20)	70	45 days or until mice death	Running in a speed-regulated treadmill (max. 13 m/min), for 30 min, 5 days/week	↔ Onset of disease ↔ Survival ↔ Motor performance
Desseille et al. (65)	M (*n* = 18)	70	45 days	Running in a speed-regulated treadmill (max. 13 m/min), for 30 min, 5 days/week	↔ Glucose tolerance ↔ Plasma lactate ↔ GLUT4 expression ↔ Lipid synthesis

*M, male; F, female; GLUT4, glucose transporter type 4; statistically significant increase (↑), decrease (↓) and no statistically significant changes (↔) compared to sedentary/no-exercising SOD1^G93A^ ALS mice*.

**Table 2:** Summary of the effects of swimming-based endurance training in SOD1^G93A^ ALS mice.

**Study**	**Sex**	**Age (days)**	**Duration**	**Exercise protocol**	**Various outcomes**
Deforges et al. (64)	M (*n* = 25)	70	45 days or until mice death	Swimming in an adjustable-flow swimming pool (max. 5 l/min), for 30 min, 5 days/week	↑ Onset of disease ↑ Survival ↑ Motor performance
Desseille et al. (65)	M (*n* = 18)	70	45 days	Swimming in an adjustable-flow swimming pool (max. 5 l/min), for 30 min, 5 days/week	↑ Glucose tolerance ↑ Plasma lactate ↑ GLUT4 expression ↑ Lipid synthesis
Flis et al. (85)	M (*n* = 8) [M (*n* = 6)—survival study]	70	45 days	Swimming in an adjustable-flow swimming pool (max. 5 l/min), for 30 min, 5 days/week for the first 35 days, and then 3 days/week in the last 10 days.	↑ Survival ↓ Mitochondrial dysfunction ↓ Oxidative stress

*M, male; F, female; GLUT4, glucose transporter type 4; statistically significant increase (↑) and decrease (↓) compared to sedentary/no-exercising SOD1^G93A^ ALS mice*.

### Gender Differences in SOD1^G93A^ ALS Mice in Response to Exercise

Kirkinezos et al. (60) was the first to investigate the effect of exercise in ALS mice and concluded that 10 weeks of endurance treadmill training can significantly extend life expectancy in high-copy number SOD1^G93A^ mice. However, when male and female mice were separately analyzed, the increase in life expectancy was only statistically significant for males, implying that testosterone levels and increased muscle mass may play a protective role in ALS (60). Sex differences in response to endurance training were also reported in ALS mice by Veldink et al. (44). However, in this case, endurance treadmill training delayed the onset of disease only in the SOD1^G93A^ female mice with a low-copy number of transgenes, but not in male mice with a low-copy number (44), suggesting a possible neuroprotective effect of female sex hormones (e.g., estrogens) in ALS. Indeed, the neuroprotective role of estrogens (67, 68) has been suggested as the potential reason why the incidence in ALS is lower in women than in men (69, 70) and why this gender difference declines gradually among older ALS patients (70) when estrogen levels decrease. Considering that testosterone levels also decrease with age (71, 72), a postmenopausal drop in the male:female ratio in ALS may not be fully explained by sex hormones, but inherent limitations that characterize epidemiologic investigations of rare and very rapidly fatal diseases, such as ALS studies.

In any case, the inconsistency of two aforementioned pre-clinical studies (44, 60) could be explained by the different training regime (see Table 1) and transgene copy number [high (60) vs. low (44)] of the SOD1^G93A^ transgene in the mice. Our experience in SOD1^G93A^ mice has shown that treatment efficacy alters to a great degree when the transgene copy number is different. When Veldink et al. (44) repeated the study using only female SOD1^G93A^ mice with a high-copy number of the transgene, they observed a significant delay in total survival time in the exercised group, compared to the sedentary group. This could be associated with the fact that the sedentary SOD1^G93A^ mice (low-copy number) presented a period of anestrus (i.e., lack of a normal estrus cycle) related with low levels of estrogens (73) contrary to the counterpart exercised mice that all of them appear a normal estrus cycle (mice with anestrus: 3/10 vs. 0/10, *p* = 0.05) (44). Although estrogen levels were not presented in this study (44), as the plasma levels of estrogens were below the detection threshold of their radioimmunoassay, there are numerous studies reporting that the expression of estrogen receptors (74, 75), and serum (74) and cerebellar (76) estrogen levels increase following endurance training. High-dose treatment of 17β-estradiol, which is an estrogen steroid hormone, is suggested to delay ALS disease progression (77, 78) and rescue the lifespan (78) of ovariectomized female SOD1^G93A^. Surprisingly, histological analysis in the lumbar ventral horns of low-copy SOD1^G93A^ mice showed no significant differences in motor neuron counts and muscle fiber size between exercising and sedentary mice in both male and female animals (44). More extensive histological analysis is needed to investigate whether these results could be explained by the low transgene copy number of SOD1^G93A^ mice.

### Effects of Exercise Intensity in SOD1^G93A^ ALS Mice

The intensity of endurance training is a parameter that potentially influences the health outcome of SOD1^G93A^ mice. Carreras et al. found that moderate intensity endurance exercise significantly delayed the onset of motor performance deficit in male SOD1^G93A^ mice (copy number is not reported) (61). This delay correlated with a 2-fold higher motor neuron density in the ventral horn of the lumbar spinal cord. This observation is contrary to results of Veldink et al. (44), who found no significant differences in motor neuron counts between exercising and sedentary low-copy SOD1^G93A^ mice in both male and female after moderate-intensity endurance exercise. Although copy number is not reported by Carreras et al. (61), any mention of SOD1^G93A^ usually refers to the more common high-copy number unless it is not specified. Therefore, the potential different copy number and the different age and, as such, disease stage that mice started exercising [30 days old (61) vs. 56 days old (44)] may explain the discrepancies observed between these two studies. Given that SOD1^G93A^ mice become symptomatic approximately at 80–90 days old (79), experimental designs including exercise training, as a therapeutic approach, which starts before disease onset are not clinically relevant. Such pre-symptomatic studies can provide important information about the effects of exercise on ALS development rather than exercise as an ALS therapeutic on which this review focuses.

Although the study of Carreras et al. (61) was not designed to monitor the longevity of the mice, a trend for higher survival rate was detected among moderate-exercised SOD1^G93A^ mice compared to sedentary or high intensity-exercised SOD1^G93A^ mice (premature deaths: 5/22 moderate-exercised vs. 7/22 sedentary vs. 10/23 high intensity-exercised). In contrast, high intensity endurance exercise significantly hastened the onset of motor performance deficits in SOD1^G93A^ mice (61). In parallel with these results, Mahoney et al. (46) concluded that regular, high intensity endurance training accelerated the deficit in motor performance and reduced survival in male, but not female, SOD1^G93A^ mice. High intensity endurance training did not increase survival in female SOD1^G93A^ mice (46). These sex differences could be explained by the potential neuroprotective role of estrogens, but further investigation is needed for confirming this postulation. Surprisingly, this type of exercise did not affect the onset of clinical symptoms in either male or female SOD1^G93A^ mice (46). Based on the authors' assumptions (46), the detrimental effects of high intensity endurance training on SOD1^G93A^ can be explained either from the intense activation of antioxidant system leading to increased oxidative stress in skeletal muscle and motor neurons or from the increased demand of adenosine triphosphate leading to excessive mitochondrial activation, which disrupts mitochondrial capacity (46). Although these physiological assumptions sound reasonable considering the potential decreased capacity of SOD1^G93A^ mice to restore the exercise-induced neuromuscular damage, more studies are needed for supporting this notion. Similarly, Kassa et al. did not find any change in disease onset after performing high intensity, endurance exercise in SOD1^G93A^ mice (63). Surprisingly, they suggested that this type of exercise may exert a neuroprotective effect on motoneurons acting on glial environment, reducing astrocytic activation and activating a protective microglial phenotype in SOD1^G93A^ mice. This finding seems inconsistent, as exercise did not improve the ALS phenotype. Considering that the sample size was only 5 SOD1^G93A^ mice, these results do not provide scientifically meaningful information.

Generally, high intensity endurance exercise is considered as a more potent stimulus for antioxidant enzyme adaptation than low intensity exercise (80). For this reason, it would be expected that higher exercise intensity may further improve the ALS phenotype and physiological responses of SOD1^G93A^ mice. However, the existing studies suggest that SOD1^G93A^ mice seem more vulnerable to high intensity stress-related stimuli (46, 61). Given that this type of mouse model has mutation in a major antioxidant enzyme (i.e., SOD1), the ability of these mice to deal with increased exercise-induced reactive oxygen species (ROS) levels may be restricted. Therefore, any extrapolation of the results should be interpreted with caution.

### Effects of Exercise Duration in SOD1^G93A^ ALS Mice

The duration of physical activity may also affect the therapeutic benefit associated with endurance exercise in SOD1^G93A^ mice. Six and 12 h, but not 2 h, daily exposure to running wheels improves motor function in SOD1^G93A^ mice with a high-copy number of the transgene, with the 6 h exercise exhibiting the most beneficial effect on lifespan (62). These exercise benefits were observed in both male and female SOD1^G93A^ mice (62). In contrast, Liebetanz et al. (45) concluded that extensive endurance exercise including 10 h daily exposure to running wheels in either gender had no significant effect on the clinical onset and progression of disease or in the lifespan of SOD1^G93A^ mice (copy number is not reported) (45). However, exercised SOD1^G93A^ mice showed an increase in their survival by 1 week compared to the sedentary counterparts, although it was not statistically significant (45). Considering that 1 week in the age of mice corresponds to ~1 year of life for humans when correlating their entire lifespan (81) and that the median survival of ALS patients is between 2 and 4 years from the onset of disease (1), the 1 week improvement in SOD1^G93A^ mice lifespan could be biologically significant, even if it did not reach the statistical significance (*p* < 0.05). In any case, the sample size (*n* = 12, 5 females/7 males) used in this study (45) is unlikely underpowered given previous evidence shown through power calculations that *n* = 8 SOD1^G93A^ mice per group are sufficient to give a significant difference in lifespan means (*p* = 0.05) with 80% power (82).

Table 1 provides an overview of the effects of exercise intensity and duration, as well as the effects of gender in response to exercise in SOD1^G93A^ mice.

### Effects of Exercise Mode (Running vs. Swimming) in SOD1^G93A^ ALS Mice

#### Skeletal Muscle Improvements in Response to Swimming

Another type of endurance training investigated in SOD1^G93A^ mice is swimming. Swimming-based training using an adjustable-flow swimming pool can significantly delay the disease onset and extend the mean survival in male SOD1^G93A^ mice compared to sedentary male SOD1^G93A^ mice (64). Surprisingly, no improvement was found with running-based training in the same study (64). Kirkinezos et al. who followed exactly the same training program, found a significant increase in the lifespan of male SOD1^G93A^ mice after running-based training (60). Considering that both studies used a similar sample size of male SOD1^G93A^ mice, the inconsistency of these results may be explained by the different time point that mice started exercising [49 day (60) vs. 70 days (64)]. In any case, findings of pre-clinical studies could have practical applications in clinical conditions only if exercise starts after disease onset. Moreover, only swimming-based training efficiently preserved the muscle phenotype including the number of myofibers, their cross-section area and distribution, in the ALS mice to the extent that it was similar in morphology to the muscles of the control group. Specifically, swimming limited the ALS-induced hypotrophy in both slow- and fast- twitch muscles, as well as maintaining the fast phenotype in fast-twitch muscles (64).

#### Metabolic Improvements in Response to Swimming

Improvements in glucose metabolism in the SOD1^G93A^ mice after swim training have also been reported (65). Specifically, swimming-based training restored impaired glucose tolerance in late symptomatic SOD1^G93A^ mice (65). On the other hand, running-based training had more modest effects (65). The benefits of swimming-based training were related to changes in skeletal muscle energetic metabolism of SOD1^G93A^ mice, shifting energetic fuels to the anaerobic glycolytic pathway (65). This metabolic shift was associated with an enhanced triglyceride storage in skeletal muscle and reduced the dysregulated expression of autophagic molecules such as Bcl2, Becn1, LC3b, and Sqstm1 (P62) (65). In case of huge energetic demands, autophagy can play a crucial role in skeletal muscle metabolic balance involving the degradation of cellular intrinsic components to produce the three main energetic macromolecules, glucose, lipids and amino acids (83). Given that ALS mice are characterized by an excessive lipid catabolism (84), glucose re-use and fat deposition induced by the swimming-based training are considered beneficial adaptations in SOD1^G93A^ mice. The beneficial role of swimming in ALS energetic metabolism is confirmed by Flis et al. who found improvements in skeletal muscle energy metabolism, oxidative stress, and mitochondrial and endoplasmic reticulum membrane modeling and function in SOD1^G93A^ mice after training in a swimming pool with an adjustable flow (85). The swim training prolonged the lifespan of SOD1^G93A^ mice with accompanying changes including increased caveolin-1, a key regulator of cholesterol efflux, decreased cholesterol accumulation in the crude mitochondrial function, improved bioenergetics (cytochrome c oxidase activity, respiratory capacity ratio, lactate dehydrogenase activity) and decreased oxidative stress.

#### Neuroprotective Improvements in Response to Swimming

Swimming also exhibited neuroprotective effects and improved motor function with delayed spinal motoneuron death and preservation of motoneurons with large soma area (64). In contrast, a dramatic motoneuron loss and an increased proportion of motoneurons with small soma area were observed after running-based training. Additionally, reduced astrogliosis and apoptosis of oligodendrocytes were evident in the spinal cord of SOD1^G93A^ mice after swimming-based training (64). This may be explained by the fact that swimming is associated with high hindlimb movement amplitude (cm) and frequency (cycles min^−1^) exercise, preferentially activating a sub-population of large motoneurons innervating fast motor units (86). In contrast, running is related with low hindlimb movement amplitude and frequency exercise resulting in the activation of a sub-population of small motoneurons (86). Therefore, the combined beneficial effects of swimming on muscle, neuronal tissue and glucose metabolism suggests swimming, at least in ALS SOD1^G93A^ mice, as a very encouraging exercise intervention. Table 2 provides an overview of the physiological adaptations to swimming in SOD1^G93A^ mice.

Overall, mild-to-moderate endurance training seems to have positive effects in SOD1^G93A^ mice, increasing the survival and motor performance as well as delaying the onset and progression of the disease with swimming-based endurance training perhaps the most beneficial type of exercise in ALS SOD1^G93A^ mice. Given that resistance training is suggested as a major type of exercise in many clinical cases, future studies using resistance-based exercise intervention in ALS mice are needed. Furthermore, since ALS is a disease with complex and multiple pathologic abnormalities, successful combinatorial therapeutic approaches with different but complementary mechanism of action may have a beneficial effect in ALS treatment. Scientific evidence suggests running with *ad libitum* exposure in running wheels, combined with virally-induced Insulin Growth Factor-1 overexpression, has synergistic and maximal effects on survival and motor function of SOD1^G93A^ mice, compared with either monotherapy-treated or no-treated SOD1^G93A^ mice (62). As a result, exercise training could be an adjunct therapy to other pharmacological treatments of ALS, but further investigation is needed to confirm this notion. Finally, an additional limitation is that the SOD1^G93A^ transgenic mouse, the most extensively used mouse model, is not representative of all ALS cases as mutations in the *SOD1* gene account for only 2% of all ALS, primarily familial, cases (87). In addition, *SOD1* gene is a major antioxidant enzyme and its mutation may make SOD1 mice unable to deal with any exercise-induced oxidative-stress. For these reasons, pre-clinical studies involving additional animal models based on both sporadic and familial ALS phenotype (88–90), are needed to advance our understanding of the effects of exercise in ALS.

## Effects of Exercise in ALS Patients

Several clinical studies have been performed to determine whether endurance and/or resistance exercise ameliorates symptoms and improves the health of ALS patients. Regardless of its modality, exercise seems to have beneficial effects on the quality of life of ALS patients, but its impact on survival has not been confirmed. In any case, no negative outcomes have been found in ALS progression after an exercise intervention.

### Endurance Exercise

Even though endurance exercise promotes cardiorespiratory fitness, cellular metabolism increasing mitochondrial biogenesis and intramuscular fuel stores, and psychological well-being, little is known about its effect on ALS pathophysiology. Pinto et al. (91) investigated the possibility of reducing motor decline by exercising ALS patients to the anaerobic threshold, simultaneously ensuring their respiratory insufficiency with the assistance of a non-invasive ventilator, the Bipap STD^®^. ALS patients performing a ramp treadmill exercise protocol for 1 year decreased the rate of respiratory deterioration and improved their functional independent mobility score, compared to a non-exercised control group (91). Similarly, Sanjak et al. (92) demonstrated that an 8 week walking program on a weight-supported treadmill for 30 min, three times weekly, significantly improved the ALS Functional Rating Scale (ALSFRS) score as well as tolerability, gait speed, distance and stride length during 6 min walk tests for ALS patients (92). The feasibility of performing moderate endurance training with non-invasive ventilation or body weight supporting system in ALS patients was confirmed recently where improvements in functionality and cardiorespiratory factors were found (93). The same research group also suggested that tele-monitored home-based endurance exercise consisting of 15 min walking on a treadmill or outdoors, is feasible, safe, user-friendly and had excellent compliance in ALS patients (94). Despite the absence of a non-exercise ALS control group in this study, there were no differences in cardiorespiratory factors (percentage of saturation of oxygen and force vital capacity) observed over the course of the exercise program (6 months). This implies a beneficial role of a home-based endurance training in ALS patients protecting them from the degenerative effects during disease progression. On the other hand, Clawson et al. recently reported that 24 week endurance training in both lower and upper body using a minicycle was not as well-tolerated as resistance training or stretching/range of motion exercises (95). Although the endurance training was not harmful for ALS patients in this study, it did not increase their muscle strength, functionality or quality of life. The inconsistency of these results could be explained by the different type of endurance training [cycling (95) vs. walking (91–94)] and the different muscle groups [upper and lower limbs (95) vs. only lower limbs (91–94)] activated during training sessions.

While respiratory muscle training is beyond the scope of this review, it is worth highlighting its potential to improve respiratory function, one of the major factors of aerobic/endurance training, in ALS patients. Specifically, scientific evidence shows that a 12 week inspiratory muscle training programme consisted of inhaling and exhaling through a specific device (Respironics^®^) may slow the decline in respiratory function in ALS patients through strengthening their inspiratory muscles (96). However, more detailed clinical assessment is needed as a later study using this type of training did not find either negative or positive effects on respiratory function of ALS patients (97). Furthermore, a combinatorial training including both respiratory muscle and endurance training could potentially induce positive adaptations for ALS patients, but this requires experimental validation.

Overall, endurance training with a supplemental support such as ventilation or weight support seems to have positive effects on respiratory capacity, functionality, tolerability and physical performance in ALS patients (Table 3), but further studies with a larger number of participants and inclusion of a control group are needed to confirm these findings. Given that each type of endurance training activates particular muscle groups and creates specific mechanical loading, different adaptations are induced by walking, cycling and swimming. Although swimming is suggested as an advantageous type of exercise in many neurological disorders (98–100), its beneficial role has not yet been experimentally validated in ALS patients. For this reason, further investigation is required to optimize an endurance training protocol for ALS patients.

**Table 3:** Summary of the effects of endurance training in ALS patients.

**Study**	**Sex**	**Age (years)**	**Disease duration**	**Duration**	**Exercise protocol**	**Various outcomes**
Clawson et al. (95)	M (*n* = 15) + F (*n* = 5)	58	7 months	24 weeks	10 min of upper limb exercise followed by 10 min lower limb exercise using a minicycle. 40–70% of target HR or 13–15 in Borg scale, 3 days/week	↓ Tolerability and compliance to exercise ↔ Functionality (ALSFRS score) ↔ Muscle strength ↔ VO_2_ max
Pinto et al. (91)	M (*n* = 6) + F (*n* = 2)	62	13 months	12 months	Ramp treadmill protocol. Exercise performed with the assistance of the ventilator Bipap STD®.	↓ rate of respiratory deterioration ↑Functional independent mobility
Sanjak et al. (92)	M (*n* = 3) + F (*n* = 3)	58	N/A	8 weeks	Walking on a weight-supported treadmill for 30 min (6 sets × 5 min, 5 min rest), 3 days/week	↑ Tolerability (measured by RPE) ↑ Functionality (ALSFRS score) ↑ Exercise performance (distance, speed, stride length)
Braga et al. (93)	M (*n* = 18) + F (*n* = 6)	63	11 months	6 months	Moderate aerobic exercise on a treadmill with non-invasive ventilation and body weight supporting system, 2 days/week	↑ ALSFRS total score ↔ ALSFRS total score slope ↑ VO_2_ peak
Braga et al. (94)	M(*n* = 7) + F (*n* = 3)	57	8 months	6 months	Walking 15 min with 5 min warm-up and 5 cool-down, at 75% of HR_max_, at least 1 day/week	↓ Functionality (ALSFRS score) ↓ Level of physical activity

*M, male; F, female; N/A, not available; ALSFRS, ALS functional rating scale; VO_2_ max, maximum rate of oxygen consumption; HR_max_, maximum heart rate; statistically significant increase (↑), decrease (↓) and no statistically significant changes (↔) compared to no-exercising/“usual care” treated ALS patients or compared to pre-exercise values. Age represents the average age of patients*.

### Resistance Exercise

Resistance exercise improves muscle force/power, induces muscle hypertrophy, maintains skeletal muscle function and prevents disability (101). In ALS patients, resistance training imparts protective benefits despite not reducing disease progression (Table 4). One of the first published reports of resistance training in ALS patients concluded that resistance exercise to the upper extremities can increase muscle static strength of the upper body (102). A subsequent study observed that moderate regular resistance training may have a mild, temporary positive effect on motor deficit and disability in ALS patients (103). Specifically, modest-intensity resistance exercises involving most muscle groups of the four limbs and trunk, induced significant improvements on functional and spasticity scores, but not on muscle strength, fatigue, pain and quality-of-life scores after 3 months of intervention (103). However, after 6, 9, or 12 months of resistance training, no further change was observed in these parameters. Similarly, Kitano et al. (104) recently suggested a 6 month home-based, whole-body strength and stretching exercise program as a safe mode of exercise training without adverse effects in ALS patients. This exercise program alleviated the functional deterioration in patients with early- stage ALS, but did not improve their upper and lower body muscle strength. In addition to these results, a new study showed that although 24 week resistance training using both lower and upper body is well-tolerated and safe for ALS patients, it is not sufficient to improve their muscle strength, functionality or quality of life (95).

**Table 4:** Summary of the effects of resistance training in ALS patients.

**Study**	**Sex**	**Age (years)**	**Disease duration**	**Duration**	**Exercise protocol**	**Various outcomes**
Bohannon (102)	F (*n* = 1)	56	22 months	75 days	Resistance exercises to upper extremities (2 sets × 10 reps, 5 min rest), for 6 days/week	↑ Static force in 14 muscle groups of upper extremities ↓ Static force in 4 muscle groups of upper extremities
Drory et al. (103)	M (*n* = 8) + F (*n* = 6)	58	21 months	12 months	Resistance exercises to whole body, against modest loads, lasted 15 min twice daily	↑ Functionality (ALSFRS score) at 3 months only ↔ Manual muscle strength
Kitano et al. (104)	M (*n* = 15) + F (*n* = 6)	63	2 months	6 months	Resistance training and stretching for the trunk muscles, upper and lower limbs, 6 days/week	↔ ALSFRS total score, but ↑ compared to control group ↔ Muscle strength of upper and lower body
Clawson et al. (95)	M (*n* = 9) + F (*n* = 9)	64	7 months	24 weeks	Resistance exercise in upper and lower body at 40% (week: 0–2), 50% (week: 3–4), and 70% (week: 5–24) 1RM, 3–5 s between reps, 2 min between sets, 4 min between muscle groups, 3 days/week	↔ Tolerability and compliance to exercise ↔ Functionality (ALSFRS score) ↔ Muscle strength
Bello-Haas et al. (47)	N/A (*n* = 13)	N/A	N/A	6 months	Moderate-load and moderate-intensity resistance exercise program to upper and lower extremities, 3 days/week	↑ Functionality (total and combined ALSFRS score) ↑ Quality of life (SF-36 score) ↓ Decline in leg strength
Jensen et al. (49)	M (*n* = 5) + F (*n* = 1)	62	<12 months (*n* = 5) 180 months (*n* = 1) Mean value N/A	12 weeks	Resistance exercises targeting both upper and lower body (2 sets × 5 reps at 6RM), 2–3 days/week	↔ Functionality (ALSFRS score and TUG) ↑ Functionality (measured by 30 s chair rise) ↓ Lower and upper body strength and power
Lunetta et al. (48)	N/A (*n* = 10)	18–75	≤24 months	6 months	Active exercises against gravity in six muscle groups in the upper and lower limbs (3 sets × 3 reps each muscle group) performed daily for 2 weeks each month	↔ Functionality (ALSFRS total score) ↔ Survival ↔ Respiratory function ↑ Subjective sense of well-being
	N/A (*n* = 10)	18–75	≤24 months	6 months	Passive exercises consisting of 20 min of 20 flexion–extension movements per minute in the upper and lower limbs, daily for 2 weeks/month	↔ Functionality (ALSFRS total score) ↔ Survival ↔ Respiratory function

*M, male; F, female; N/A, not available; Reps, repetitions; ALSFRS, ALS functional rating scale; SF-36, MOS 36-Item Short Form Survey; TUG, timed up and go; statistically significant increase (↑), decrease (↓) and no statistically significant changes (↔) compared to no-exercising/”usual care” treated ALS patients or compared to pre-exercise values. Age represents either the average age or the age range of patients*.

Furthermore, Lunetta et al. found that a 6 month strength training program, based on either active exercises against gravity in upper and lower limbs or passive exercises consisting of flexion-extension movements in the upper and lower limbs, cannot improve the functionality (ALSFRS total score), survival or quality of life in ALS patients, even though they were reporting subjectively an improvement in their sense of well-being at the end of every exercise session (48). On the other hand, a randomized controlled trial showed that a 6 month resistance training program with moderate intensity and moderate load involving both lower- and upper-body muscles, can induce significantly better function, less decline in leg strength, and higher quality of life in ALS patients without any adverse effects (47).

In contrast, negative outcomes in neuromuscular function of ALS patients have been found after 12 weeks of resistance training (49). The whole-body resistance training did not significantly affect functionality and average cross-section area of type I and II muscle fibers, but loss of muscle strength and power in both lower and upper body muscle groups increased following the training period (49). Also, an increase in the percentage of both atrophied and greatly (abnormal) hypertrophied type II fibers combined with a decrease in normal-sized type II fibers were observed after the training period (49). Taking into account of the small number of patients (only 5) recruited in this study (49), their heterogeneous disease progression and the study design (12 week “lead-in” control period without exercising), the findings should be interpreted with caution. In ALS studies, the use of a lead-in control period (i.e., a period with no exercise), before receiving an intervention instead of using a control, non-exercised ALS group may confound the final results. Considering the potential rapid decline in health and the immediacy of death in ALS patients, the muscle fiber atrophy and the deteriorated muscle strength after resistance training may be partly explained by the disease progression occurring during the 12 week lead-in period rather than by any detrimental effects caused by exercise.

In summary, most studies suggest that resistance training can improve the quality of life of ALS patients increasing their functionality and, in some cases, their muscle strength. However, resistance training seems to have no effect on the lifespan of ALS patients. Future attempts to restrict, as much as possible, the inherent limitations of ALS clinical studies such as the small sample size and the heterogeneity of patients are needed for confirming the existing findings. Ideally, further studies with a stronger experimental design including a greater number of participants with homogeneous disease progression would provide more valid information about the effects of resistance training in ALS patients. Unfortunately, the reality is different as there are too few patients and only a small proportion of them eventually participates in exercise clinical trials.

### Concurrent Endurance and Resistance Exercise

Concurrent training is a type of exercise that combines endurance and resistance training models and can stimulate both aerobic and anaerobic metabolic pathways, inducing a variety of beneficial adaptations. A recent randomized controlled trial suggested that a 6 month exercise program involving a combination of resistance and endurance training can reduce the motor deterioration of ALS patients (48). Specifically, the exercise training consisting of active exercises against gravity in the upper and lower limbs combined with cycle ergometer activity for 20 min increased the functionality (ALSFRS total score) of ALS patients. Furthermore, all exercised ALS patients reported subjectively an improvement in their sense of well-being at the end of every exercise session, although no improvements in their survival, respiratory function and quality of life were observed (48). However, the limited number of patients (*n* = 10, sex ratio is not reported) (48) raises concerns about the interpretation of these findings and, therefore, these results should be considered with caution. Additionally, a recent study suggested concurrent training as a feasible and beneficial intervention for ALS patients (105). Specifically, Merico et al. found that 5 week moderate, submaximal resistance and endurance training improved the functional independence, oxygen consumption, fatigue and muscle strength in ALS patients (105). The positive effects of combined endurance and resistance training in ALS patients were also confirmed in a retrospective, non-consecutive case series study (106). In this study, 2 week endurance training on a cycle ergometer combined with lower-body resistance training appeared feasible and beneficial for ALS patients improving their muscle strength at the early stage of disease but not at the late stage of disease (106). Given that initial improvements in muscle strength, especially among untrained people, are explained mainly by the increased voluntary neural activation of the trained muscles and not by muscle hypertrophy which has a gradually increasing role in strength development as the training proceeds (after the first 3 to 5 weeks) (107, 108), a longer exercise intervention (>5 weeks) could induce further and greater skeletal muscle adaptations in ALS patients at either early or late stage of disease. In addition, while encouraging, these findings should be interpreted carefully considering the lack of statistical analysis due to the very small sample size (*n* = 2) used in this study (106). An overview of the effects of concurrent endurance and resistance training in ALS patients is summarized in Table 5.

**Table 5:** Summary of the effects of concurrent endurance and resistance training in ALS patients.

**Study**	**Sex**	**Age (years)**	**Disease duration**	**Duration**	**Exercise protocol**	**Effects of exercise**
Lunetta et al. (48)	N/A (*n* = 10)	18–75	≤24 months	6 months	Active exercises against gravity in the upper and lower limbs (3 sets × 3 reps each muscle group) combined with cycle ergometer activity at 60% maximal power output, for 20 min. Performed daily for 2 weeks/month	↑ Functionality (measured by ALSFRS total score) ↔ Survival ↔ Respiratory function
Merico et al. (105)	M (*n* = 17) + F (*n* = 9)	62	30 months	5 weeks	Resistance training with submaximal isometric contractions, 3 reps with 30 s of rest between for each bilateral muscle segment, daily for 5 weeks. + Endurance training at 65% HR_max_, 15–20 min on a cycle ergometer, ergometry arm-leg and/or treadmill, based on the weakness pattern of each patient, daily for 5 weeks.	↑ Functionality (FIM) ↓ VO_2_ submax ↑ Muscle strength (MRC muscle grading scale) ↔ Muscle strength (dynamometric measures) ↔ 6 min walk test ↑ Fatigue Severity Scale (compared to the baseline, but not to the control group)
Kato et al. (106)	M (*n* = 2)	1st case: 60 2nd case: 52	1st and 2nd case: 1st intervention: 10 and 15 months; 2nd intervention: 20 months for both	1st and 2nd case: 2 weeks for each intervention	Both cases: 1st and 2nd intervention: lower-body resistance training using weights and machines, at 5 score of Borg scale (lower limbs), combined with endurance training on a bicycle ergometer, respiratory exercises and gait exercises	1st and 2nd case: 1st intervention: ↑ knee extension muscle strength ↔ and ↑ Functionality (FAC score) 2nd intervention: ↔ knee extension muscle strength ↔ Functionality (FAC score)

*M, male; F, female; N/A, not available; Rep, repetitions; ALSFRS, ALS functional rating scale; VO_2_ submax, oxygen uptake at submaximal work; HR_max_, maximum heart rate; FIM, Functional Independence Measure; MRC, medical research council; FAC, functional ambulation categories; statistically significant increase (↑), decrease (↓) and no statistically significant changes (↔) compared to no-exercising/”usual care” treated ALS patients or compared to pre-exercise values. Age represents either the average age or the age range of patients*.

Given the inherent methodological limitations involving clinical studies of rare and rapidly fatal diseases, such as ALS studies, further investigation with sufficient cohort size, limited variability of ALS causality, less heterogeneity in the disease stage of patients and adequate control of confounders is needed for confirming the existing findings of endurance, resistance, and concurrent training clinical studies in ALS patients. In addition, experimental designs including control group and proper statistical analysis are important for concluding to valid results. Also, future studies involving alternative training modes with less risk of muscle damage and injuries, such as swimming, could provide useful information to conclude to an optimal exercise program for ALS patients. Recent pre-clinical studies suggest swimming (64, 65, 85) as the most beneficial type of exercise in ALS, but clinical studies are needed to translate these mouse exercise results to humans.

## Conclusions

Although previous evidence suggests a potential relationship between heavily active lifestyles and an increased incidence of ALS (30, 31, 109), recent studies support that physical activity is not necessarily a risk factor for ALS (42, 43, 110). There is now a large body of evidence suggesting physical activity as a potential therapeutic or even holistic approach for ALS.

Pre-clinical mouse studies conclude that the intensity, duration and mode of exercise, as well as gender, can influence health outcomes in ALS progression as identified in SOD1^G93A^ mice in response to regular long-term endurance training (44, 61, 62, 64). Findings from this review suggest that mild-to-moderate endurance training is a potential beneficial exercise intervention for ALS SOD1^G93A^ mice (44, 60, 61), in particular, swimming (64, 65, 85). However, many of these pre-clinical studies are characterized by limitations related to variable sample sizes, heterogeneous transgene copy number or a non-clinically-relevant timing of intervention. An additional limitation is the lack of pre-clinical studies focused on the effect of resistance-based exercise, a major type of exercise, in ALS. A current *in vivo* study shows that repeated sessions of isometric tetanic contractions, which represent a resistance-based exercise protocol for experimental animals, can improve contractile function and ameliorate distinct histopathological features of skeletal muscle in Duchenne muscular dystrophy circumventing the concern of potentially injurious eccentric contractions (111). A similar resistance-based exercise program including non-injurious isometric contractions could also have positive effects in skeletal muscle of ALS mice. Further pre-clinical studies are needed to investigate the effects of exercise not only *per se* but also as an adjunct therapy to other pharmacological treatments of ALS. Finally, except for the SOD1^G93A^ transgenic mouse, which represents only the 2% of all, primarily familial, cases, additional animal models based on both sporadic and familial ALS phenotype (88–90) are needed to advance our understanding of the effects of exercise in ALS.

Despite mild-to-moderate exercise training improving the survival of ALS mice, currently, there is no clinical evidence supporting this notion in ALS patients (48). Regardless, most clinical studies suggest that every type of exercise training including stretching, resistance, endurance or concurrent training, has an advantageous impact on the quality of life of ALS patients increasing mainly their functionality (47, 48, 91–93, 103, 105) and sometimes their muscle strength (105, 106) and/or their cardiorespiratory function (91, 93, 105). The discrepancy between preclinical and clinical findings could be partly explained by the limitations observed in most of clinical studies in ALS, such as a small sample size, non-representative control populations, inadequate control of confounders and heterogeneous disease stage of patients.

Importantly, the effect of swimming has not yet been experimentally validated in ALS patients, although exercising in water is commonly recommended for patients with other neurological disorders such as Parkinson's disease and multiple sclerosis (98–100). Given that swimming movements mainly incorporate concentric muscle contraction, a swim-based training may protect skeletal muscles of ALS patients from the high muscle stress induced by eccentric muscle contraction experienced with other types of exercise, such as running or resistance training. Furthermore, the water minimizes biomechanical stress on muscles and joints decreasing the risk of muscle damages and injuries. As water itself creates resistance to movement, performing exercises in water has been suggested as an alternative training mode to improve neuromuscular conditioning in healthy population (112, 113). Considering all these aspects, swimming with supplemental support or aquatic exercise training could be a potential exercise treatment for ALS patients.

While the benefits of exercise in ALS patients are still not clear, nor have the effects of swimming yet been determined, there appears promise ahead for further studies investigating the therapeutic benefits of exercise, which should be supported by well-designed and statistically powered, pre-clinical studies in multiple rodent models of ALS. In addition to the physical impact, the holistic value of exercise may also be key to the improved well-being of ALS patients.

## Author Contributions

All authors listed have made a substantial, direct and intellectual contribution to the work, and approved it for publication.

### Conflict of Interest Statement

The authors declare that the research was conducted in the absence of any commercial or financial relationships that could be construed as a potential conflict of interest.
